# Novel insights into mechanisms of inhibition of colonic motility by loperamide

**DOI:** 10.3389/fnins.2024.1424936

**Published:** 2024-08-29

**Authors:** Nabil Parkar, Nick J. Spencer, Luke Wiklendt, Trent Olson, Wayne Young, Patrick Janssen, Warren C. McNabb, Julie E. Dalziel

**Affiliations:** ^1^AgResearch, Palmerston North, New Zealand; ^2^Riddet Institute, Massey University, Palmerston North, New Zealand; ^3^School of Food and Advanced Technology, Massey University, Palmerston North, New Zealand; ^4^College of Medicine and Public Health, Flinders Health and Medical Research Institute, Adelaide, SA, Australia

**Keywords:** opiate, peristalsis, enteric nervous system, loperamide, constipation

## Abstract

**Background:**

It is well known that opiates slow gastrointestinal (GI) transit, via suppression of enteric cholinergic neurotransmission throughout the GI tract, particularly the large intestine where constipation is commonly induced. It is not clear whether there is uniform suppression of enteric neurotransmission and colonic motility across the full length of the colon. Here, we investigated whether regional changes in colonic motility occur using the peripherally-restricted mu opioid agonist, loperamide to inhibit colonic motor complexes (CMCs) in isolated mouse colon.

**Methods:**

High-resolution video imaging was performed to monitor colonic wall diameter on isolated whole mouse colon. Regional changes in the effects of loperamide on the pattern generator underlying cyclical CMCs and their propagation across the full length of large intestine were determined.

**Results:**

The sensitivity of CMCs to loperamide across the length of colon varied significantly. Although there was a dose-dependent inhibition of CMCs with increasing concentrations of loperamide (10 nM - 1 μM), a major observation was that in the mid and distal colon, CMCs were abolished at low doses of loperamide (100 nM), while in the proximal colon, CMCs persisted at the same low concentration, albeit at a significantly slower frequency. Propagation velocity of CMCs was significantly reduced by 46%. The inhibitory effects of loperamide on CMCs were reversed by naloxone (1 μM). Naloxone alone did not change ongoing CMC characteristics.

**Discussion:**

The results show pronounced differences in the inhibitory action of loperamide across the length of large intestine. The most potent effect of loperamide to retard colonic transit occurred between the proximal colon and mid/distal regions of colon. One of the possibilities as to why this occurs is because the greatest density of mu opioid receptors are located on interneurons responsible for neuro-neuronal transmission underlying CMCs propagation between the proximal and mid/distal colon. The absence of effect of naloxone alone on CMC characteristics suggest that the mu opioid receptor has little ongoing constitutive activity under our recording conditions.

## Introduction

Embedded within the gut wall lies a complex neural circuitry containing millions of neurons that are critical for the modulation of gastrointestinal (GI) functions including motility and control of fluid movement. This autonomous neural system that has the unique ability to coordinate GI functions independent of the central nervous system (CNS) is referred to as the enteric nervous system (ENS) (Furness, 2012; Furness et al., 2014). Control of GI functions is dependent on the activity of diverse neurochemical classes of enteric neurons that are contained within interconnected micro-ganglia that form two distinct plexi, known as the submucosal and myenteric plexus (Spencer and Hu, 2020). The myenteric plexus lies between the circular and longitudinal muscle layers of the gut wall and includes excitatory and inhibitory motor neurons, descending and ascending interneurons and a unique population of intrinsic sensory neurons which are responsible for coordinating GI motor activity enabling propulsive movement along the intestine (Wood, 2012; Spencer et al., 2021). The unique ENS characteristic of regulating intestinal movements independently of the CNS allows for the exploration of enteric neuronal networks and their role in facilitating motility in isolated segments of intestine (Furness et al., 1995).

An important aspect of digestion is the controlled progression of luminal contents along the GI tract, which is coordinated by various patterns of intestinal movements that occur as a result of the interplay among enteric neural circuits, Interstitial cells of Cajal (ICC) and spontaneous intestinal smooth muscle activity (Costa and Furness, 1982; Huizinga et al., 1998, 2014). Colonic motor complexes (CMCs) are neurally mediated spontaneous propagating contractions of the colonic smooth muscle that propagate over longer distances and aid in the propulsion of luminal contents (Corsetti et al., 2019). They can be readily recorded and analyzed from isolated intact colon preparations as they are known to occur in the absence of input from the CNS. The mouse colon has served as an excellent model species for recording CMCs because of the regular and rhythmic occurrence of these events in the isolated colon (Fida et al., 1997; Bush et al., 2000; Roberts et al., 2007). They are the predominant mechanical motor pattern of the mouse colon and are visualized as rhythmic contractions that propagate along at least half the total length of the colon (Spencer and Bywater, 2002).

Opioids have been used for many years as anti-diarrheal agents that work by inhibiting enteric neuronal activity, reducing propulsive colonic peristaltic contractions, and delaying GI transit (Holzer, 2009). Several studies have reported exogenous opioids to alter intestinal motility both via *in vivo* and *in vitro* settings (Tavani et al., 1980; Smith et al., 2012; Dalziel et al., 2016; Gade et al., 2016). The impact of opioids on GI motility is ascribed to the activation of opioid receptors (μ/mu, κ/kappa, and δ/delta), with the mu opioid receptor playing a significant role in mediating anti-peristaltic effects specifically in the large intestine (Galligan and Sternini, 2016). Loperamide is one such opioid agonist that acts on mu opioid receptors decreasing the activity of the myenteric plexus within the ENS which in turn reduces the tone of the longitudinal and circular smooth muscles of the intestine inhibiting peristaltic activity (Van Nueten et al., 1979; Mellstrand, 1987). In the guinea pig ileum, loperamide has been shown to act via mu opioid receptors to inhibit excitatory motor neurons of the ENS (Waterman et al., 1992). Moreover, loperamide has shown to inhibit acetylcholine release, the primary excitatory neurotransmitter released by myenteric neurons to induce muscle contractions, by interacting with opiate receptor sites in the myenteric plexus (Yagasaki et al., 1978). Although there is detailed knowledge about the pharmacological activity of loperamide, little is known of its effects on the characteristics of colonic motility patterns in *ex vivo* colon. This is surprising and is an obvious gap in knowledge, despite it being a commonly used drug to treat diarrhea. The current study assays the effect of loperamide on colonic motor complexes in isolated colon of mice using high resolution spatio-temporal diameter maps (D-Maps). Here we uncovered major new clues as to how and where mu opioid receptors predominantly act to retard colonic transit.

## Materials and methods

### Animals

The research described in this paper was carried out in compliance with the Animal Welfare Act, 1999 (NZ) and after obtaining ethical approval from the AgResearch Grasslands Animal Ethics Committee (Palmerston North, New Zealand) under application AE 15540. C57BL/6 mice were bred at The University of Otago breeding unit (Dunedin, New Zealand) and were raised in groups with their littermates. Mice were kept in controlled conditions, with a consistent temperature of 21°C and a regular light/dark cycle (06:00/18:00). They were housed in cages lined with sawdust, either made of plastic or stainless steel, and had unrestricted access to food and water. Animal weight, food intake, and general health were monitored daily using a scoring system ranging from 1 to 5, following the NZ Animal Health Care Standard.

### Drugs and solutions

The composition of Krebs solution used was (in mM): NaCl. 118; KCl. 4.7; NaHPO_4_.2H_2_O. 1.0; NaHCO_3_. 25; MgCl.6H_2_O. 1.2; D-Glucose. 11; CaCl_2_.2H_2_O. 2.5. The pH of Krebs solution was maintained at 7.3–7.4 with constant aeration of 95% O_2_ and 5% CO_2_ at 35 ± 0.5 °C.

The drugs used in these experiments included loperamide and naloxone that were purchased from Selleck Chemicals (Houston, TX, USA). Both loperamide and naloxone were prepared as stock solutions in, dimethyl sulfoxide (DMSO) and MilliQ water (based on solubility), respectively. Final concentrations of the drugs were made using Krebs solution (the final concentration of the drugs was calculated based on the organ bath tank volume). The final DMSO concentration was 0.1% which was also used for the control.

### Dissection protocol and colonic tissue preparation

**FIGURE 1:**
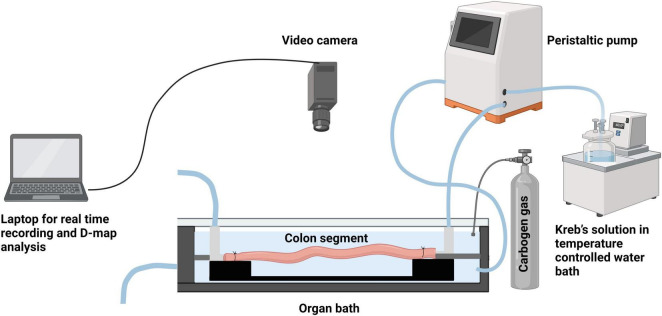
Schematic representation of the *ex vivo* colonic motility experiment set up. (Created using BioRender).

Male mice aged between 7 and 14 weeks were humanely euthanized by isoflurane overdose inhalation followed by cervical dislocation, following approved protocols from the AgResearch Grasslands Animal Ethics Committee. The mouse was securely fixed to a dissection board by pinning its four paws, enabling the exposure of its ventral side. Using dissecting forceps and scissors, an incision was made through the epidermis, revealing the lower abdominal muscle layers. The abdominal cavity was opened along the midline towards the sternum. To prevent tissue dehydration during the dissection, Krebs solution at room temperature, aerated with carbogen gas (95% O_2_ and 5% CO_2_), was poured onto the abdominal contents at regular intervals (every 30–60 s). The surrounding tissues, such as the urinary bladder and testes, were carefully removed. Two vertical incisions, approximately 0.5 cm from the midline, were made to cut the pelvic bone on each side of the colon. The colon, still attached to the caecum and rectum, was isolated. Using forceps, the caecum was held while the proximal end of the colon was separated from it. Next, the entire length of the colon was placed in a glass tray filled with warm Krebs solution (30–35°C), which was continuously bubbled with carbogen. Fine dissection scissors were used to trim the mesentery attached to the tissue. Care was taken not to stretch the gastrointestinal tissue while trimming the adjoining mesentery. To empty the intestinal contents and faecal pellets, the isolated colon was kept in Krebs solution and constantly bubbled with carbogen for 30 min. Any remaining contents were flushed out by gently applying pressure at the oral end using a 5 ml syringe attached to a blunt needle filled with Krebs solution. A stainless-steel rod (1.9 mm thickness) was gently inserted into the full length of the colon lumen. The proximal and distal ends of the colon were secured onto barbed tubing connectors and secured with surgical knots. The tubing connectors were anchored to a base plate, which could be easily transferred into an organ bath. The setup, including the isolated colon segment with the stainless-steel rod, was then placed into an organ bath containing warm Krebs solution (maintained at 35 ± 0.5°C) and continuously aerated with carbogen (Figure 1). The lumen of the colon was perfused with warm Krebs solution at a rate of 0.13 mL min^–1^ from the proximal end using a peristaltic pump. This constant flow provided the necessary pressure for recording consistent propagating contractions. The distal end of the colon, attached to the tubing connector, was cannulated to an outlet tube. Intraluminal pressure was measured by determining the difference in height between the end of the outlet tube and the meniscus of the Krebs solution within the organ bath, maintained at a constant level of 40–50 mm. The organ bath chamber was continuously perfused with Krebs buffer using a peristaltic pump.

### Study design

Motility experiments began with a 30 min equilibration period, followed by a 10 min control video recording. In specific experiments, loperamide was either applied to the organ bath in isolation from any antagonists or combined with the opioid antagonist naloxone. In other experiments, naloxone was added on its own. In each experiment where the tissue was exposed to pharmacological agents, motility was recorded for 10 min. Each tissue preparation served as its own control prior to drug application. Pharmacological agents were applied to the serosal side of each preparation, along the length of the bath gently with a pipetter. We first applied increasing concentrations of loperamide (10 nM, 100 nM and 1 μM) with 10 min of continuous bath perfusion to washout in between each, to establish an effective partial inhibitory concentration. This concentration was then used directly in subsequent experiments.

### Video Imaging and analysis

Colonic contractions or motility patterns were recorded *ex vivo* using a camera (Logitech HD Pro C920; JB Hi-Fi, New Zealand) mounted directly above the organ bath. QuickTime (Apple Inc.) software was used to record videos. The captured video segments (10 min duration) were analyzed using MATLAB (MathWorks, Natick, MA, USA) to create spatiotemporal maps (ST) where the diameter of the colon is mapped (displayed as a heat map) along the length of the segment as a function of time. The x axis represents increasing time (s), and the y axis represents length of colon (in mm). A color bar indicated luminal diameter where relaxed tissue is represented by blue–green pixels on the ST maps while yellow–red pixels represented constricted regions. Four parameters of colonic motor activity were examined: CMC propagating velocity, CMC interval, CMC propagation distance and the frequency of CMCs. CMCs, defined as contractions which originated at the oral end and propagated aborally at least half the length of the colon, were analyzed and from these CMC propagating velocity, CMC interval and CMC propagation distance were assessed. The frequency of CMCs was quantified from ST maps while the velocity of CMC propagation, interval between CMCs and CMC propagation distance were measured using MATLAB. The CMC propagation velocity was determined using the scale produced with each map (showing distance and time) to determine over what distance of the colon the contraction occurred and the time it took for the full contraction to take place. Interval between CMCs was calculated as the average time between contractions at a point that was selected as a consistent reference location for comparing different preparations.

### Statistical analysis

Statistical analysis was performed using GraphPad Prism software (version 9.5.1, GraphPad software Inc., USA). Differences in CMC parameters between multiple treatment groups were assessed using Kruskal-Wallis ANOVA with Dunn’s multiple comparison test. Results are expressed as mean ± standard error of mean (SEM). *P*-value less than 0.05 was considered statistically significant.

## Results

### General observations

After euthanasia and inspection of the abdominal cavity, it was established that each of the 20 colonic preparations had an average of 3 faecal pellets within the lumen (range: 1–5). After placing each preparation containing pellets into the glass tray containing warm Krebs (30–35°C; aerated with carbogen gas), it was found that 7 of these 20 preparations naturally expelled all pellets within 30 min. Four colon preparations did not expel any pellets. All colon preparations were gently flushed with approximately 5 ml of warm Krebs solution. After an initial equilibrium period (30 min), CMC typically became temporally coordinated between the proximal and distal ends of the isolated colon.

### Effect of loperamide on CMC parameters

**FIGURE 2:**
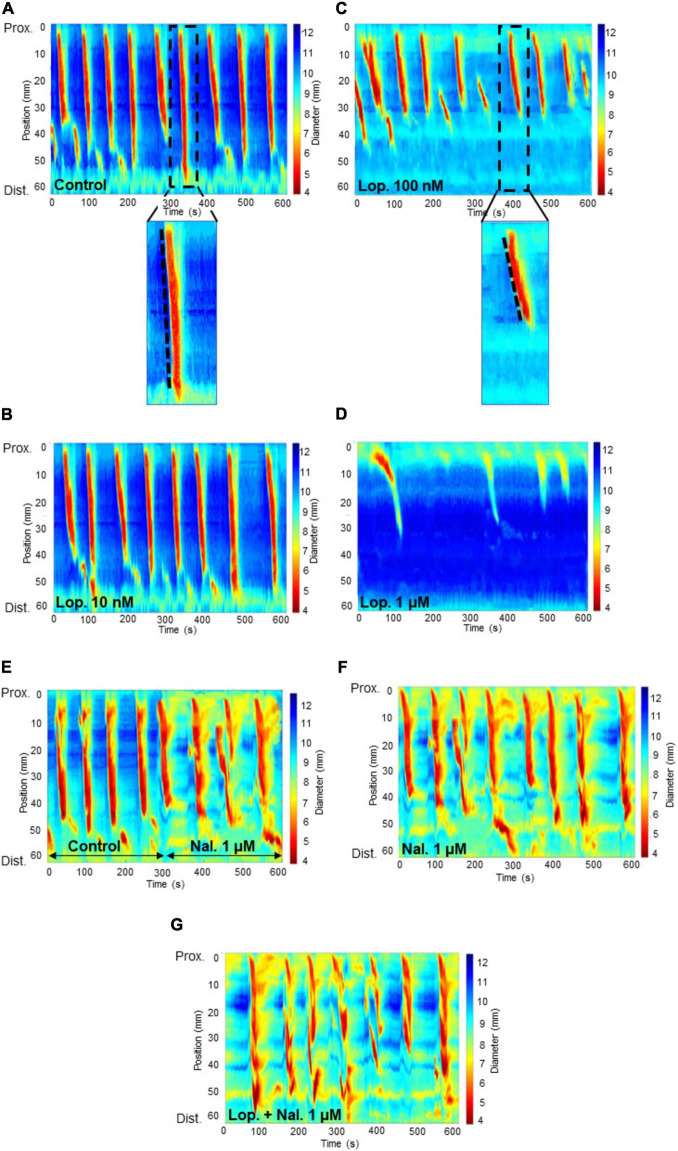
Spatiotemporal heat maps (D-Maps) showing colonic motor complexes (CMCs) with treatments applied in the order: from one experiment: **(A)** control **(B)** loperamide at 10 nM concentration **(C)** loperamide at 100 nM concentration **(D)** loperamide at 1 μM concentration, and from another experiment: **(E)** control for 5 min, then naloxone 1 μM concentration for 5 min (in the same recording) **(F)** naloxone at 1 μM concentration **(G)** naloxone at 1 μM added to the bath containing preparations which were exposed to loperamide at 100 nM concentration. The x-axis represents increasing time in seconds and the y-axis represents length of colon in millimeters from the proximal to distal end. The color bar on the right of each map indicates the width of the colon for each captured frame during the 10 min video recording. Red-yellow regions show constricted areas whereas blue-green regions show relaxed tissue. The broken lines within the enlarged images of the spatiotemporal maps in **(A)** and **(B)** show the velocity of the CMCs. The less steep the slope, the slower the CMC velocity. Lop: loperamide; nal: naloxone; Prox: proximal; Dist: distal.

**FIGURE 3:**
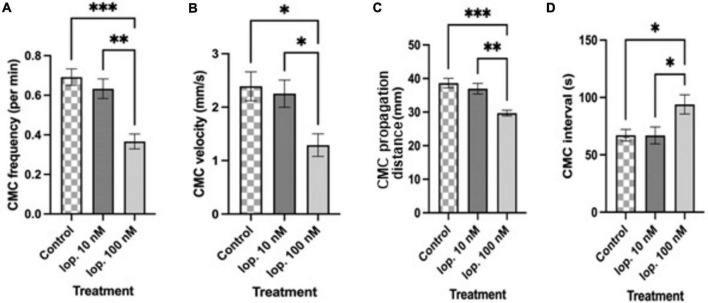
Summary graphs of different treatment effects on **(A)** colonic motor complex (CMC) frequency per min **(B)** CMC velocity (mm/s) **(C)** CMC propagation distance (mm) and **(D)** CMC interval (s). Loperamide at 10 nM (Lop. 10 nM) concentration was without significant effect on CMC parameters. Loperamide at an increased concentration of 100 nM (Lop. 100 nM) significantly reduced CMC frequency, CMC velocity and CMC propagation distance compared to controls. CMC intervals increased with lop. 100 nM compared to controls. Loperamide at 1 μM had a strong inhibitory effect (but CMCs did not meet the criteria of propagating at least half the length of the colon so are not shown here). Asterisks indicate statistical significance (*P < 0.05; **P < 0.01; ***P < 0.001). Data shown as mean with error bars indicating SEM. CMC, colon motor complex; lop, loperamide.

To assess the effect of mu opioid receptor agonist loperamide on CMC activity, we characterized the changes in interval between CMCs and the velocity with which CMCs propagated along the colon (Figures 2A–D). The effect of increasing loperamide concentration on CMC parameters was analyzed (Figures 3A–D). It was found that loperamide 10 nM did not significantly alter any of the CMC parameters (Figures 2A, B, 3A–D).

Loperamide at 100 nM significantly reduced CMC frequency compared to the control period (control: 0.69 ± 0.04 min^–1^; lop. 100 nM: 0.36 ± 0.03 min^–1^; *n* = 12; *P* = 0.0001) (Figures 2C, 3A). Moreover, this concentration significantly reduced CMC velocity by 46% compared to the control period (control: 2.39 ± 0.27 mm s^–1^; lop. 100 nM: 1.28 ± 0.21 mm s^–1^; *n* = 12; *P* = 0.0103) (Figures 2C, 3B). Similarly, the extent of CMC propagation was significantly reduced when 100 nM loperamide was added to the bath (control: 38.60 ± 1.42 mm; lop. 100 nM: 29.70 ± 0.84 mm; *n* = 12; *P* = 0.0002) (Figure 3C). Also, the interval between CMCs increased by 40% when 100 nM loperamide was applied (control: 67.12 ± 5.06 s; lop. 100 nM: 93.97 ± 8.36 s; *n* = 12; *P* = 0.0299) (Figure 3D). Loperamide at 1 μM potently inhibited CMCs in the mid and distal colon (Figures 2D, 3A–D) but did not completely block CMCs in the proximal colon. These contractions propagated for short distances (less than 50%; Lop. 1 μM: 0.35 ± 0.02 min^–1^; n = 4). These partial contractions were excluded from further analysis as they did not meet the criteria of propagating at least half the length of the colon.

### Effect of naloxone in the presence of loperamide

**FIGURE 4:**
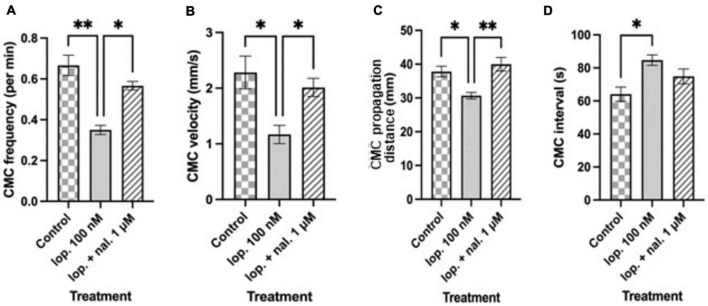
Summary graphs of different treatment effects on **(A)** colonic motor complex (CMC) frequency per min **(B)** CMC velocity (mm/s) **(C)** CMC propagation distance (mm) and **(D)** CMC interval (s). Loperamide at 100 nM (lop. 100 nM) significantly reduced CMC frequency, CMC velocity and CMC propagation distance compared to controls. CMC intervals increased with lop. 100 nM compared to controls. Naloxone at 1 μM prevented the inhibitory effects of loperamide, except for CMC intervals, when applied to preparations that were exposed to 100 nM loperamide (lop. + nal. 1 μM). Asterisks indicate statistical significance (*P < 0.05; **P < 0.01; ***P < 0.001). Data shown as mean with error bars indicating SEM. CMC: colonic motor complex; lop: loperamide; nal: naloxone.

Naloxone at 1 μM was used to test selectivity of loperamide for opiate receptors and whether the inhibitory actions of loperamide could be reversed. Naloxone (1 μM) was added to the bath containing preparations which were exposed to loperamide at 100 nM concentration (Figures 2E–G). Loperamide significantly reduced CMC frequency compared to control activity, and this effect was prevented by naloxone when co-applied with loperamide (lop. 100 nM: 0.35 ± 0.02 min^–1^; lop. + nal. 1 μM: 0.56 ± 0.02 min^–1^; *n* = 6; *P* = 0.0377), which was not significantly different from controls (Figure 4A). Similarly, the reduced velocity of CMCs after loperamide application was restored by naloxone (lop. 100 nM: 1.16 ± 0.16 mm s^–1^; lop. + nal. 1 μM: 2.01 ± 0.16 mm s^–1^; *n* = 6; *P* = 0.0174), to similar levels as controls (Figure 4B). CMC propagation distance which was significantly reduced with 100 nM loperamide was also restored by naloxone to distances similar to controls (lop. 100 nM: 30.67 ± 0.97 mm; lop. + nal. 1 μM: 39.98 ± 1.99 mm; *n* = 6; *P* = 0.0061) (Figure 4C). The interval between CMCs increased significantly in the presence of loperamide and this effect was partially prevented by naloxone (lop. 100 nM: 84.71 ± 3.17 s; lop. + nal. 1 μM: 74.89 ± 4.52 s; *n* = 6; *P* = 0.4323) (Figure 4D).

To confirm that there was no intrinsic effect caused by naloxone or the presence of endogenous opioids, naloxone was added alone to the organ bath. Naloxone at 1 μM did not affect CMC parameters as compared to the control period (Figures 2E, F).

## Discussion

We identified major regional differences of inhibition of colonic motility with the peripherally restricted mu receptor agonist, loperamide. The most significant result of this study was that loperamide never blocked CMCs in the proximal colon but had a major inhibitory effect in the mid and distal colon. The results revealed that loperamide 100 nM increased the interval between CMCs in the proximal colon and decreased the velocity of CMCs. This effect was dose dependent, because loperamide 10 nM did not have the same effects. The attenuation of inhibitory effects of loperamide on CMCs by naloxone confirmed the involvement of opioid receptor signaling.

As previously classified, colonic contractions that migrated at least half the length of the colon were termed as CMCs (Corsetti et al., 2019). At baseline or control conditions, CMCs occurred at a frequency of 0.69 min^–1^ which is similar to previous mouse studies, where CMCs in control conditions have been shown to occur at a frequency of 0.5–3 min^–1^ (Fida et al., 1997; Brierley et al., 2001; Roberts et al., 2008; Spencer et al., 2013). However, the techniques used to stimulate colonic preparations to record CMC activity in these studies were different from the current study. With regards to the velocity of propagation of CMCs, the findings of this study were found to be similar to a study by Balasuriya and colleagues (Balasuriya et al., 2016) who used a similar technique to record CMCs. That is, maintaining colonic distension via infusion of intraluminal fluid that acted as stimuli enabling video recordings of CMC activity. CMCs require gut wall distension in mouse colon and are infrequent in the absence of an applied mechanical stimulus (Barnes et al., 2014). A study by Barnes and colleagues showed that the frequency with which CMCs occurred was highly dependent upon the level of stretch applied to the colon, but were absent or occurred rarely when the colon was devoid of endogenous faecal pellets (Barnes et al., 2014). The mechanism by which initiation of content dependent neural peristalsis and of CMCs have been investigated in studies using mouse colon *in vitro*. Keating and Spencer showed that the neural circuits responsible for CMC generation lie in the myenteric plexus and/or muscularis externa (Keating and Spencer, 2010). Moreover, the removal of the mucosa and submucosal plexus did not prevent CMC propagation along the colon, although the characteristics of CMC propagation were altered (Keating and Spencer, 2010). These findings suggest that the submucosal plexus and mucosa are not essential for either the initiation or propagation of CMCs. In another study, Zagorodnyuk and Spencer demonstrated that stretch applied on the luminal wall increases CMC frequency which remains unaffected even after removal of mucosa and submucosa (Zagorodnyuk and Spencer, 2011). Overall, this demonstrated that all the intrinsic neural apparatus necessary to generate CMCs lies in the myenteric plexus and/or muscularis externa. It is possible that myogenic pacemaker cells (interstitial cells of Cajal, ICC) could contribute to CMC characteristics. An analogy is the small intestine, where migrating motor complexes (MMCs) still occur in *W/W*^v^ mutant mice that lack pacemaker type ICC-MY (and electrical slow waves), but MMC characteristics are slightly different (Spencer et al., 2003).

The findings of the current study strongly suggest the actions of loperamide act via suppression of cholinergic neurotransmission in the myenteric plexus. Suppression of GI motor activity is a well-known effect of opioids based on studies involving animals and humans (Tavani et al., 1980; Wintola et al., 2010; Dalziel et al., 2016; Heitmann et al., 2022). In the present study, loperamide at a relatively higher concentration suppressed CMC parameters compared to controls. This is similar to a study by Beckett and colleagues who in their experiments showed that morphine, another mu opioid receptor agonist, reduced the amplitude and frequency of CMCs in isolated mouse colon compared to control conditions (Beckett et al., 2018). The inhibition of GI motor activity by exogenous opioids such as loperamide and morphine is largely due to the existence of a widely distributed opioid system in the gut (Sternini et al., 2004). Opioid receptors of the mu, kapa and delta-subtypes are expressed by myenteric and secretomotor neurons of the ENS in rodents, guinea pigs and humans, but with a varied distribution across GI regions and species (Bagnol et al., 1997; Ho et al., 2003; Gray et al., 2006; Poole et al., 2011; Sobczak et al., 2013). Gut motility is controlled by myenteric neurons via the release of neurotransmitters acetylcholine (ACh) and substance P which induce muscle contraction (excitatory) and adenosine triphosphate/β-nicotinamide adenine dinucleotide (ATP/βNAD), nitric oxide (NO), vasoactive intestinal peptide (VIP) to cause muscle relaxation (inhibitory) (Brookes, 2001). The inhibitory effect of mu opioid agonists have been shown to arise primarily from interruption of both excitatory and inhibitory enteric neural inputs controlling muscle activity (Yagasaki et al., 1978; Waterman et al., 1992; Nishiwaki et al., 2000; Iwata et al., 2007). A study by Yagasaki and colleagues using isolated guinea pig ileum demonstrated that exposure to loperamide results in suppression of ACh and substance P release (Yagasaki et al., 1978). Since the predominant input to longitudinal muscle is excitatory, the absence of ACh and substance P result in inhibition of longitudinal muscle contraction. In another study Iwata and colleagues using electrophysiological techniques elucidated that contraction in circular muscle of the isolated mouse ileum induced by morphine administration was strongly inhibited by NG-nitro-L-arginine and tetrodotoxin, suggesting that morphine’s contractile effects on circular muscle may be associated with the inhibition of NO release from inhibitory nerves (Iwata et al., 2007). In a state of rest, the continuous firing of inhibitory motor neurons leads to the relaxation of the circular muscle layer. Consequently, this relaxation of the bowel aids in accommodating advancing intraluminal contents. However, when exposed to an opioid agonist, the suppression of NO, as demonstrated by Iwata and colleagues, triggers heightened contractile activity within this muscle layer, inhibiting the descending relaxation necessary for peristalsis. Thus, the inhibition of circular muscle relaxation would contribute to loperamide’s ability to suppress propulsive motility patterns (i.e., CMCs).

We also observed that colonic relaxation was not always complete at higher concentrations of loperamide and note that it is not completely without untargeted actions, for example 1 μM inhibits the large conductance calcium-activated potassium (BK) ion channel (Vouga et al., 2021). While cell membrane permeability to access the internal site to act as a state-dependent pore blocker would be very low, such an effect would be expected to increase cellular excitability and thereby decrease relaxation, increasing muscle tone and contribute to the slightly higher resting tone observed. Naloxone applied alone did not alter CMC frequency or duration compared with controls, but some additional effects were observed on the relaxation phase that affected colonic diameter. This may have been an additional inhibitory effect on any basal constitutive activity of opioid receptors. Further studies will be needed to determine the neuronal types involved in pharmacological responses.

A major finding of this study was that loperamide reduced the extent of propagation of CMCs. This occurred via a suppression of CMC contraction propagation from proximal to mid and distal regions of colon. In control conditions CMCs propagated longer distances, frequently reaching the distal end of the colon. However, when loperamide was applied, this effect was potently reduced. A possible explanation for these findings is that opiate receptors activated by loperamide are either more densely distributed on interneurons and motor neurons in the mid to distal colon, or that a similar opiate receptor density exists throughout the ENS, but the receptors have a greater sensitivity to opiate agonists between the mid to distal regions. This finding could contribute to explaining why constipation is common in people who frequently consume opiates. Future studies could investigate whether differences in the relative distribution of mu opioid receptors might underlie the regional changes detected in relation to inhibition of CMCs by loperamide. Further insight on possible sex differences could be explored by verifying whether colonic motility responds similarly to loperamide in female mice and should measure estrus status to account for any hormone related variations in analyses.

## Conclusion

The findings show that loperamide inhibited CMCs in a dose dependent manner and preferentially between the mid and distal colon. These results provide key insights into the regional effects of opioid inhibition of colonic motility.

## Data Availability

The raw data supporting the conclusions of this article will be made available by the authors, without undue reservation.

## References

[B1] Bagnol D. Mansour A. Akil H. Watson S. (1997). Cellular localization and distribution of the cloned Mu and kappa opioid receptors in rat gastrointestinal tract. *Neuroscience* 81 579–591. 10.1016/s0306-4522(97)00227-3 9300443

[B2] Balasuriya G. Hill-Yardin E. Gershon M. Bornstein J. C. (2016). A sexually dimorphic effect of cholera toxin: Rapid changes in colonic motility mediated via a 5-HT3receptor-dependent pathway in female C57BL/6 mice. *J. Physiol*. 594 4325–4338. 10.1113/JP272071 26990461 PMC4967745

[B3] Barnes K. Beckett E. Brookes S. Sia T. Spencer N. (2014). Control of intrinsic pacemaker frequency and velocity of colonic migrating motor complexes in mouse. *Front. Neurosci.* 8:96. 10.3389/fnins.2014.00096 24847200 PMC4021129

[B4] Beckett E. Staikopoulos V. Hutchinson M. (2018). Differential effect of morphine on gastrointestinal transit, colonic contractions and nerve-evoked relaxations in toll-like receptor deficient mice. *Sci. Rep.* 8 5923. 10.1038/s41598-018-23717-4 29651005 PMC5897409

[B5] Brierley S. Nichols K. Grasby D. Waterman S. (2001). Neural mechanisms underlying migrating motor complex formation in mouse isolated colon. *Br. J. Pharmacol.* 132 507–517. 11159701 10.1038/sj.bjp.0703814 PMC1572567

[B6] Brookes S. (2001). Classes of enteric nerve cells in the Guinea-pig small intestine. *Anat. Record* 262 58–70. 11146429 10.1002/1097-0185(20010101)262:1<58::AID-AR1011>3.0.CO;2-V

[B7] Bush T. Spencer N. Watters N. Sanders K. Smith T. (2000). Spontaneous migrating motor complexes occur in both the terminal ileum and colon of the C57BL/6 mouse in vitro. *Auton. Neurosci.* 84 162–168. 10.1016/S1566-0702(00)00201-0 11111848

[B8] Corsetti M. Costa M. Bassotti G. Bharucha A. Borrelli O. Dinning P. (2019). First translational consensus on terminology and definitions of colonic motility in animals and humans studied by manometric and other techniques. *Nat. Rev. Gastroenterol. Hepatol.* 16 559–579. 10.1038/s41575-019-0167-1 31296967 PMC7136172

[B9] Costa M. Furness J. (1982). Nervous control of intestinal motility. *Med. Drugs GastrointesT. Motil.* 1982 279–382.

[B10] Dalziel J. Young W. Bercik P. Spencer N. Ryan L. Dunstan K. (2016). Tracking gastrointestinal transit of solids in aged rats as pharmacological models of chronic dysmotility. *Neurogastroenterol. Motil.* 28 1241–1251. 10.1111/nmo.12824 27028044

[B11] Fida R. Lyster D. Bywater R. Taylor G. (1997). Colonic migrating motor complexes (CMMCs) in the isolated mouse colon. *Neurogastroenterol. Motil.* 9 99–107. 9198085 10.1046/j.1365-2982.1997.d01-25.x

[B12] Furness J. (2012). The enteric nervous system and neurogastroenterology. *Nat. Rev. Gastroenterol. Hepatol.* 9 286–294. 10.1038/nrgastro.2012.32 22392290

[B13] Furness J. Callaghan B. Rivera L. Cho H. (2014). The enteric nervous system and gastrointestinal innervation: Integrated local and Central Control. *Adv. Exp. Med. Biol.* 2014 39–71. 10.1007/978-1-4939-0897-4_3 24997029

[B14] Furness J. Johnson P. Pompolo S. Bornstein J. (1995). Evidence that enteric motility reflexes can be initiated through entirely intrinsic mechanisms in the guinea-pig small intestine. *Neurogastroenterol. Motil*. 7 89–96. 10.1111/j.1365-2982.1995.tb00213.x 7621324

[B15] Gade A. Kang M. Khan F. Grider J. Damaj M. Dewey W. (2016). Enhanced sensitivity of α3β4 nicotinic receptors in enteric neurons after long-term morphine: Implication for opioid-induced constipation. *J. Pharmacol. Exp. Therap.* 357 520–528. 27068812 10.1124/jpet.116.233304 PMC4885510

[B16] Galligan J. Sternini C. (2016). Insights into the role of opioid receptors in the GI tract: Experimental evidence and therapeutic relevance. *Gastrointest. Pharmacol.* 239 363–378. 10.1007/164_2016_116 28204957 PMC6310692

[B17] Gray A. Coupar I. White P. (2006). Comparison of opioid receptor distributions in the rat ileum. *Life Sci.* 78 1610–1616. 10.1016/j.lfs.2005.07.048 16289621

[B18] Heitmann P. Keightley L. Wiklendt L. Wattchow D. Brookes S. Spencer N. (2022). The effects of loperamide on excitatory and inhibitory neuromuscular function in the human colon. *Neurogastroenterol. Motil.* 34 e14442. 10.1111/nmo.14442 36054796

[B19] Ho A. Lievore A. Patierno S. Kohlmeier S. Tonini M. Sternini C. (2003). Neurochemically distinct classes of myenteric neurons express the mu-opioid receptor in the guinea pig ileum. *J. Comp. Neurol.* 458 404–411. 10.1002/cne.10606 12619074

[B20] Holzer P. (2009). Opioid receptors in the gastrointestinal tract. *Regul. Peptid.* 155 11–17. 10.1016/j.regpep.2009.03.012 PMC3163293 19345246

[B21] Huizinga J. Ambrous K. Der-Silaphet T. (1998). Co-operation between neural and myogenic mechanisms in the control of distension-induced peristalsis in the mouse small intestine. *J. Physiol.* 506 843–856. 10.1111/j.1469-7793.1998.843bv.x 9503342 PMC2230746

[B22] Huizinga J. Chen J. Zhu Y. Pawelka A. McGinn R. Bardakjian B. (2014). The origin of segmentation motor activity in the intestine. *Nat. Commun.* 5 3326. 10.1038/ncomms4326 24561718 PMC4885742

[B23] Iwata H. Tsuchiya S. Nakamura T. Yano S. (2007). Morphine leads to contraction of the ileal circular muscle via inhibition of the nitrergic pathway in mice. *Eur. J. Pharmacol.* 574 66–70. 10.1016/j.ejphar.2007.06.029 17632101

[B24] Keating D. Spencer N. (2010). Release of 5-hydroxytryptamine from the mucosa is not required for the generation or propagation of colonic migrating motor complexes. *Gastroenterology* 138 .e1–.e2. 10.1053/j.gastro.2009.09.020 19782081

[B25] Mellstrand T. (1987). Loperamide—an opiate receptor agonist with gastrointestinal motility effects. *Scand. J. Gastroenterol.* 22 65–66. 10.3109/00365528709091001 2820051

[B26] Nishiwaki H. Saitoh N. Nishio H. Takeuchi T. Hata F. (2000). Possible role of potassium channels in MU-receptor-mediated inhibition and muscarinic autoinhibition in acetylcholine release from myenteric plexus of Guinea pig ileum. *Jap. J. Pharmacol.* 82 343–349. 10.1254/jjp.82.343 10875755

[B27] Poole D. Pelayo J. Scherrer G. Evans C. Kieffer B. Bunnett N. (2011). Localization and regulation of fluorescently labeled Delta opioid receptor, expressed in enteric neurons of Mice. *Gastroenterology* 141 982–991. 10.1053/j.gastro.2011.05.042 21699782 PMC4429902

[B28] Roberts R. Bornstein J. Bergner A. Young H. (2008). Disturbances of colonic motility in mouse models of Hirschsprung’s disease. *Am. J. Physiol. Gastrointest. Liver Physiol.* 294 4. 10.1152/ajpgi.00558.2007 18276829

[B29] Roberts R. Murphy J. Young H. Bornstein J. (2007). Development of colonic motility in the neonatal mouse-studies using spatiotemporal maps. *Am. J. Physiol. Gastrointest. Liver Physiol.* 292 G930–G938. 10.1152/ajpgi.00444.2006 17158255

[B30] Smith T. Grider J. Dewey W. Akbarali H. (2012). Morphine decreases enteric neuron excitability via inhibition of sodium channels. *PLoS One*. 7:0045251. 10.1371/journal.pone.0045251 23028881 PMC3448635

[B31] Sobczak M. Sałaga M. Storr M. Fichna J. (2013). Physiology, signaling, and pharmacology of opioid receptors and their ligands in the gastrointestinal tract: Current concepts and future perspectives. *J. Gastroenterol.* 49 24–45. 10.1007/s00535-013-0753-x 23397116 PMC3895212

[B32] Spencer N. Bywater R. (2002). Enteric nerve stimulation evokes a premature colonic migrating motor complex in mouse. *Neurogastroenterol. Motil.* 14 657–665. 12464088 10.1046/j.1365-2982.2002.00367.x

[B33] Spencer N. Hu H. (2020). Enteric nervous system: sensory transduction, neural circuits and gastrointestinal motility. *Nat. Rev. Gastroenterol. Hepatol.* 17 338–351. 32152479 10.1038/s41575-020-0271-2 PMC7474470

[B34] Spencer N. Nicholas S. Sia T. Staikopoulos V. Kyloh M. Beckett E. (2013). By what mechanism does ondansetron inhibit colonic migrating motor complexes: Does it require endogenous serotonin in the gut wall? *Neurogastroenterol. Motil.* 25 677–685. 10.1111/nmo.12136 23593931

[B35] Spencer N. Sanders K. Smith T. (2003). Migrating Motor Complexes do not require electrical slow waves in the mouse small intestine. *J. Physiol.* 553 881–893. 14514874 10.1113/jphysiol.2003.049700 PMC2343631

[B36] Spencer N. Travis L. Wiklendt L. Costa M. Hibberd T. Brookes S. (2021). Long range synchronization within the enteric nervous system underlies propulsion along the large intestine in mice. *Commun. Biol.* 4 2485. 10.1038/s42003-021-02485-4 34376798 PMC8355373

[B37] Sternini C. Patierno S. Selmer I. Kirchgessner A. (2004). The opioid system in the gastrointestinal tract. *Neurogastroenterol. Motil.* 16 3–16. 10.1111/j.1743-3150.2004.00553.x 15357847

[B38] Tavani A. Bianchi G. Ferretti P. Manara L. (1980). Morphine is most effective on gastrointestinal propulsion in rats by intraperitoneal route: Evidence for local action. *Life Sci.* 27 2211–2217. 10.1016/0024-3205(80)90386-0 7207014

[B39] Van Nueten J. Helsen L. Michiels M. Heykants J. (1979). Distribution of loperamide in the intestinal wall. *Biochem. Pharmacol.* 28 1433–1434. 444310 10.1016/0006-2952(79)90450-7

[B40] Vouga A. Rockman M. Yan J. Jacobson M. Rothberg B. (2021). State-dependent inhibition of BK channels by the opioid agonist loperamide. *J. Gen. Physiol.* 153 834. 10.1085/jgp.202012834 34357374 PMC8352719

[B41] Waterman S. Costa M. Tonini M. (1992). Modulation of peristalsis in the Guinea-pig isolated small intestine by exogenous and endogenous opioids. *Br. J. Pharmacol.* 106 1004–1010. 10.1111/j.1476-5381.1992.tb14448.x 1356564 PMC1907666

[B42] Wintola O. Sunmonu T. Afolayan A. (2010). The effect of Aloe ferox mill. in the treatment of loperamide-induced constipation in wistar rats. *BMC Gastroenterol.* 10:95. 10.1186/1471-230X-10-95 20723249 PMC2931457

[B43] Wood J. (2012). Cellular neurophysiology of enteric neurons. *Physiol. Gastrointest. Tract*. 2012 629–669. 10.1016/b978-0-12-382026-6.00021-x

[B44] Yagasaki O. Suzuki H. Sohji Y. (1978). Effects of loperamide on acetylcholine and prostaglandin release from isolated guinea pig ileum. *Jap. J. Pharmacol.* 28 873–882. 10.1254/jjp.28.873 745310

[B45] Zagorodnyuk V. Spencer N. (2011). Localization of the sensory neurons and mechanoreceptors required for stretch-evoked colonic migrating motor complexes in mouse colon. *Front. Physiol.* 2:98. 10.3389/fphys.2011.00098 22203805 PMC3244083

